# Carnosine Attenuates the Development of both Type 2 Diabetes and Diabetic Nephropathy in BTBR *ob/ob* Mice

**DOI:** 10.1038/srep44492

**Published:** 2017-03-10

**Authors:** Thomas Albrecht, Maaike Schilperoort, Shiqi Zhang, Jana D. Braun, Jiedong Qiu, Angelica Rodriguez, Diego O. Pastene, Bernhard K. Krämer, Hannes Köppel, Hans Baelde, Emile de Heer, Alessandra Anna Altomare, Luca Regazzoni, Alessandra Denisi, Giancarlo Aldini, Jacob van den Born, Benito A. Yard, Sibylle J. Hauske

**Affiliations:** 1 Department of Nephrology, Endocrinology and Rheumatology, Fifth Department of Medicine, Medical Faculty Mannheim of the University of Heidelberg, Mannheim, Germany; 2 The Department of Pathology, Leiden University Medical Centre, Leiden, the Netherlands; 3 The Department of Pharmaceutical Sciences, University of Milan, Milan, Italy; 4 Department of Nephrology, University Medical Center Groningen, Groningen, the Netherlands

## Abstract

We previously demonstrated that polymorphisms in the carnosinase-1 gene (CNDP1) determine the risk of nephropathy in type 2 diabetic patients. Carnosine, the substrate of the enzyme encoded by this gene, is considered renoprotective and could possibly be used to treat diabetic nephropathy (DN). In this study, we examined the effect of carnosine treatment *in vivo* in BTBR (Black and Tan, BRachyuric) *ob/ob* mice, a type 2 diabetes model which develops a phenotype that closely resembles advanced human DN. Treatment of BTBR *ob/ob* mice with 4 mM carnosine for 18 weeks reduced plasma glucose and HbA1c, concomitant with elevated insulin and C-peptide levels. Also, albuminuria and kidney weights were reduced in carnosine-treated mice, which showed less glomerular hypertrophy due to a decrease in the surface area of Bowman’s capsule and space. Carnosine treatment restored the glomerular ultrastructure without affecting podocyte number, resulted in a modified molecular composition of the expanded mesangial matrix and led to the formation of carnosine-acrolein adducts. Our results demonstrate that treatment with carnosine improves glucose metabolism, albuminuria and pathology in BTBR *ob/ob* mice. Hence, carnosine could be a novel therapeutic strategy to treat patients with DN and/or be used to prevent DN in patients with diabetes.

The global prevalence of type 2 diabetes is steadily increasing and has reached epidemic proportions^1^. Patients with type 2 diabetes have a 40% risk to develop diabetic nephropathy (DN), the foremost cause of end-stage renal disease (ESRD) in the Western world^2^. Despite multifactorial intervention, current therapy regimens only retard the progression of DN, but do not prevent ESRD^3^. In fact, patients with type 2 diabetes undergoing state-of-the-art treatment may still progress to ESRD, whereas others will never develop DN regardless of metabolic control^4^. This dichotomy casts doubts on our current understanding of the pathophysiology of DN and highlights the importance of genetic factors predisposing to DN^5^.

A growing body of evidence indicates a genetic contribution of the *CNDP1* gene to the development of DN^6^,^7^. This gene encodes carnosinase-1, a circulating enzyme that degrades the dipeptide carnosine. We have previously demonstrated, that patients with gene polymorphisms associated with lower serum carnosinase levels are less susceptible to the development of nephropathy. This indicates a protective role of carnosine on the kidney. Carnosine exhibits potent anti-glycation properties and was shown to protect human podocytes and mesangial cells under hyperglycemic conditions^6^,^8^. *In vivo* studies demonstrated beneficial effects of carnosine on metabolic control in rodent models of diabetes^9^,^10^. Yet, these studies were not able to show prevention of diabetic nephropathy, as the models employed do not reflect advanced stages of human DN^11^. Moreover, usage of streptozotocin-rats (STZ) is questionable as the CNDP1 association with DN is restricted to type 2 diabetes^6^.

In this study, we used the BTBR (Black and Tan, BRachyuric) *ob/ob* (leptin-deficient) mouse model to study the effect of carnosine supplementation on histopathological and molecular parameters of DN. The rapid onset and reversibility of morphologically advanced DN make this model uniquely suited for interventional studies^11^,^12^. The BTBR mouse strain is hyperinsulinemic and predisposed to obesity^13^,^14^. Mutant mice lacking the hormone leptin (*ob/ob*) are mildly hyperglycemic and develop pronounced obesity^15^. When the BTBR background is combined with the *ob/ob* mutation, mice are insulin resistant and hyperglycemic, and rapidly develop a phenotype that closely resembles advanced human DN^11^,^13^. We investigated whether carnosine supplementation could affect the development and progression of the advanced renal phenotype in BTBR *ob/ob* mice.

## Results

### Carnosine improves diabetes in BTBR *ob/ob* mice

**Figure 1:**
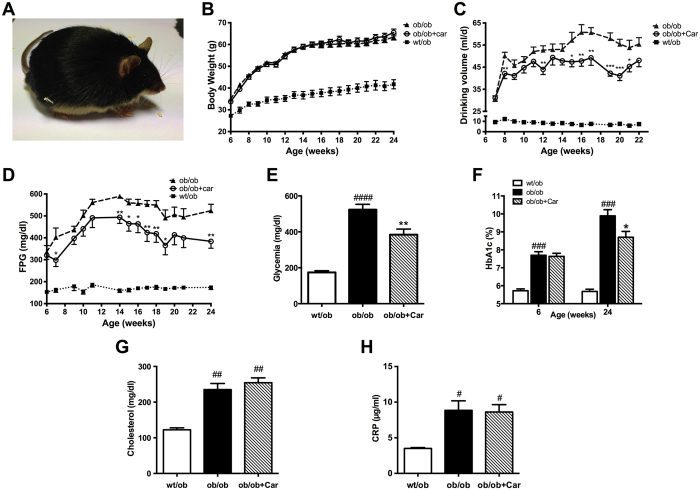
Carnosine attenuates diabetes in BTBR *ob/ob* mice. (**A**) Representative image of a 24-week-old BTBR *ob/ob* mouse. (**B**) Body weight increased in *ob/ob* mice, independent of carnosine treatment. (**C**) Daily water intake was increased in *ob/ob* mice relative to *wt/ob* mice, and attenuated in carnosine-administered animals. (**D**) Weekly determination of fasting plasma glucose (FPG) indicated manifest hyperglycemia in *ob/ob* mice, which was reduced in carnosine-treated animals throughout the observation period. (**E**) Random glycemia (measured before perfusion) was significantly lower in carnosine-administered *ob/ob* mice compared with *ob/ob* controls. (**F**) At week 24 of age (18 weeks of treatment), HbA1c levels of *ob/ob* animals were elevated, and significantly reduced in carnosine treated mice. (**G** and **H**) Serum cholesterol and C-reactive protein (CRP) (at week 24 of age) were elevated in *ob/ob* mice, and unaffected by carnosine. Data represents means ± SEM. *P < 0.05, **P < 0.01, ***P < 0.001 compared to *ob/ob* mice. ^#^*P* < 0.05, ^##^*P* < 0.01, ^###^*P* < 0.001, ^####^*P* < 0.0001 compared to *wt/ob* mice. Car: carnosine.

The BTBR *ob/ob* mouse model (Fig. 1A) has been described as a model of advanced obesity-related diabetes due to a lack of the hormone leptin. We assessed the course of diabetes over 18 weeks in three groups (*wt/ob, ob/ob*, carnosine-supplemented *ob/ob*). Three *ob/ob* mice died before the end of the study, most likely caused by their diabetic phenotype. Of these mice, two were untreated and one was treated with carnosine. As expected, homozygous mice developed obesity and increased in body weight accordingly throughout the whole observation period (Fig. 1B). In contrast, heterozygous mice increased in body weight according to expectations for non-obese healthy mice. As a surrogate for osmotic diuresis, daily water intake was assessed. Drinking volume was much higher in homozygous mice than in heterozygous mice (Fig. 1C). Interestingly, carnosine-administered *ob/ob* mice showed a significantly lower daily water intake towards the end of the experimental period compared with *ob/ob* control mice (*P* < 0.001). FPG was significantly elevated in BTBR *ob/ob* mice when compared with *wt/ob* mice. Carnosine treatment resulted in markedly reduced FPG (Fig. 1D*, P* < 0.01) compared to *ob/ob* mice and random glycemia measured before sacrifice (*P* < 0.01) (Fig. 1E). Improvement of glucose metabolism by carnosine was also confirmed by determination of glycated hemoglobin (HbA1c) (Fig. 1F). At the beginning of the observation, *ob/ob* mice already showed significantly increased HbA1c when compared to *wt/ob* mice (*P* < 0.0001). Baseline HbA1c before treatment did not differ between both diabetic groups. However, after 18 weeks of carnosine administration we observed a significant reduction in HbA1c in the treatment group when compared to *ob/ob* control mice (*P* < 0.05). Since dyslipidemia is associated with diabetes and necessary for the diagnosis of metabolic syndrome, we measured cholesterol at the end of the experiment. Serum cholesterol was significantly higher in homozygous mice (*P* < 0.01) (Fig. 1G) but not affected by carnosine administration. As a sensitive marker of inflammation, C-reactive protein (CRP) was determined in serum (Fig. 1H). Homozygous mice had significantly higher CRP when compared to *wt/ob* mice (*P* < 0.05). Carnosine administration had no effect on CRP.

### Carnosine stimulates insulin, but not glucagon secretion

**Figure 2:**
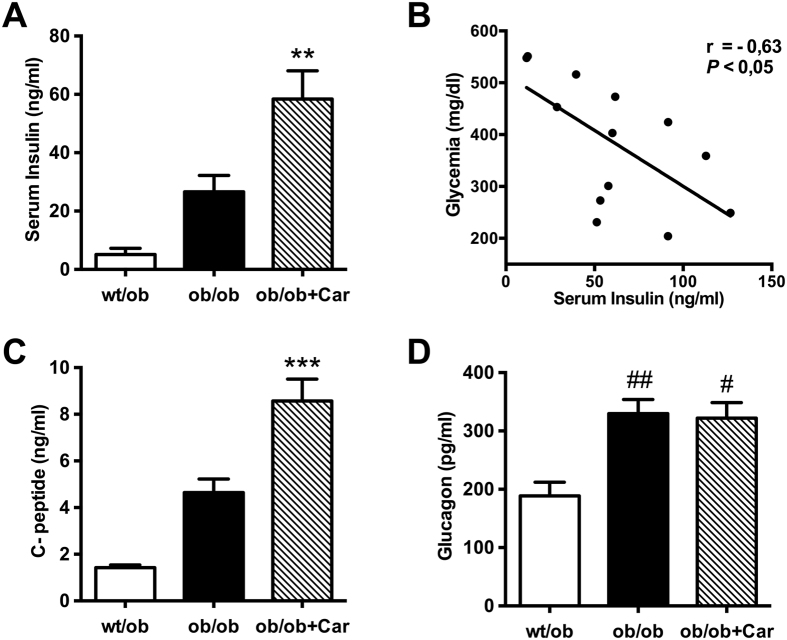
Carnosine stimulates insulin secretion. (**A**) Serum insulin levels (at week 24 of age) indicated hyperinsulinemia in *ob/ob* mice, which was further increased by more than twofold in carnosine-treated mice. (**B**) Glycemia correlated negatively with serum insulin levels in carnosine-administered mice. (**C**) C-peptide levels were significantly higher in carnosine-treated mice as compared to untreated *ob/ob* mice. (**D**) Glucagon levels were elevated in both *ob/ob* groups, but not affected by carnosine treatment. **P < 0.01, ***P < 0.001 compared to *ob/ob* mice. ^#^*P* < 0.05, ^##^*P* < 0.01 compared to *wt/ob* mice. Car: carnosine.

Being a key factor in all types of diabetes, we determined insulin levels in serum obtained at the end of the observation period (Fig. 2A). We discovered elevated serum insulin in *ob/ob* mice when compared to heterozygous controls. Interestingly, carnosine treatment further raised insulin levels significantly more than two-fold (*P* < 0.01), possibly accounting for the improved metabolic control. Indeed, we found a significant negative correlation of serum insulin and glycemia within the carnosine-administered *ob/ob* group (*P* < 0.05) (Fig. 2B). The correlation between insulin and HbA1c did not reach statistical significance. No correlation was found between insulin and the ACR. The carnosine-induced increase in serum insulin was paralleled by a significant elevation of serum C-peptide levels when compared to the *ob/ob* control group (*P* < 0.001) (Fig. 2C). Glucagon levels were elevated in both *ob/ob* groups (*P* < 0.05) (Fig. 2D), however, carnosine treatment did not affect those hormone levels.

### Carnosine attenuates albuminuria and glomerular hypertrophy

**Figure 3:**
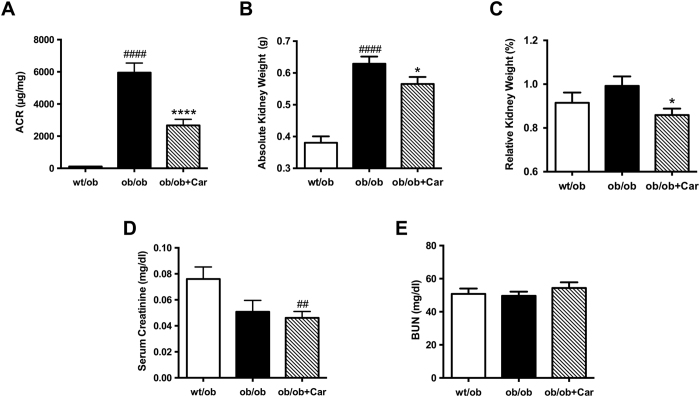
Carnosine protects from diabetic kidney damage. (**A**) Carnosine administration for 18 weeks significantly reduced the ACR at week 24 by more than twofold compared with the *ob/ob* controls. (**B**) At the end of the experiment, kidney weight (average of both kidneys) was significantly increased in *ob/ob* mice, which was attenuated in carnosine-treated mice. (**B** and **C**) At the end of the experiment, the absolute kidney weight (average of both kidneys) was significantly increased in *ob/ob* mice. Carnosine treatment resulted in both reduced absolute and relative kidney weights. (**D** and **E**) Serum creatinine and blood urea nitrogen (BUN) were determined at the end of the experiment. The highest serum creatinine levels were found in heterozygous mice, being significantly different from the lowest levels found in carnosine-supplemented homozygous mice. No differences were observed with respect to BUN between any of the groups. Data represents means ± SEM. *P < 0.05, ****P < 0.0001 compared to *ob/ob* mice. ^##^*P* < 0.01, ^####^*P* < 0.0001 compared to *wt/ob* mice. Car: carnosine.

We determined the albumin/creatinine ratio (ACR) to assess progression of diabetic nephropathy (Fig. 3A). At week 10, no significant differences in ACR were observed between treated and non-treated *ob/ob* mice, whereas at the end of the observation period the ACR was reduced remarkably in carnosine-treated mice when compared to diabetic control mice (*P* < 0.0001), which developed a 50-fold increase in ACR. To assess renal hypertrophy, kidneys were weighed after sacrifice. Absolute kidney weight was significantly higher in *ob/ob* mice when compared to *wt/ob* mice (*P* < 0.0001) (Fig. 3B). Due to the markedly lower body weights of heterozygous animals, relative kidney weights did not differ significantly from those of *ob/ob* mice (Fig. 3C). Carnosine treatment resulted in significantly reduced absolute and relative kidney weight when compared to *ob/ob* control mice (*P* < 0.05). Renal function was evaluated by serum creatinine (Fig. 3D) and blood urea nitrogen (BUN) (Fig. 3E) levels at the end of the experiment. In fact, the highest serum creatinine levels were found in heterozygous mice, being significantly different from the lowest levels found in carnosine-supplemented mice (*P* < 0.01). This difference may be explained by the higher muscle content in non-diabetic mice and is in line with other studies^11^. The higher serum creatinine levels in *wt/ob* are in line with previous studies^11^ and can be attributed to increased creatinine production in *wt/ob* mice due to a higher muscle mass and glomerular hyperfiltration occurring in *ob/ob* mice as already described^16^,^17^ and as indicated by the enlargement of Bowman’s space. None of the groups showed creatinine levels indicative for renal failure. No significant differences between treated and non-treated homozygous mice were observed with respect to both serum creatinine and BUN.

**Figure 4:**
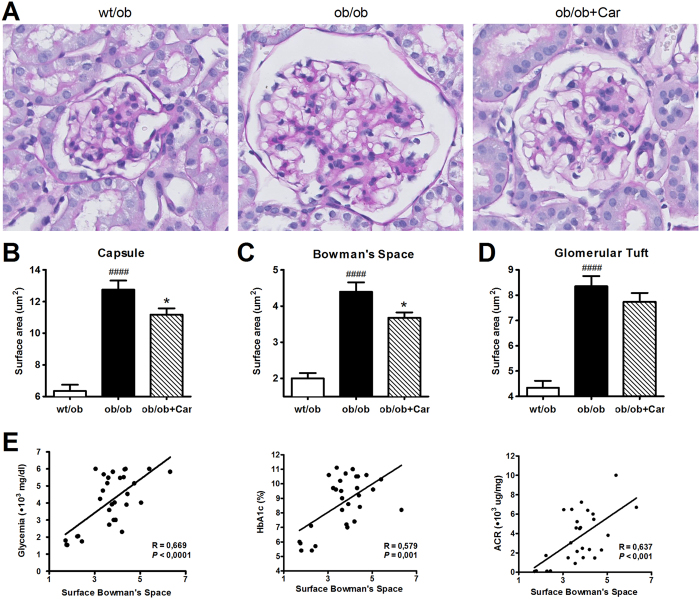
Carnosine reduces glomerular hypertrophy. (**A**) Representative images of glomeruli (PAS-stained sections) from *wt/ob, ob/ob* and *ob/ob* carnosine-supplemented mice. (**B,C** and **D**) Surface areas of Bowman’s capsule (**B**), Bowman’s space (**C**) and the glomerular tuft (**D**) were increased in *ob/ob* mice, while carnosine treated mice showed a reduction in the area of Bowman’s capsule and space. (**E**) Surface area of Bowman’s space positively correlated with glycemia, HbA1c and albumin-creatinine ratio (ACR). Data represents means ± SEM. *P < 0.05 compared to *ob/ob* mice. ^####^*P* < 0.0001 compared to *wt/ob* mice. Car: carnosine.

At the histological level, changes in surface area of the glomeruli were quantified for all groups as DN results in distinct structural alterations of the kidney (Fig. 4). BTBR *ob/ob* mice showed a significant enlargement of Bowman’s capsule (*P* < 0.0001) (Fig. 4B), Bowman’s space (*P* < 0.0001) (Fig. 4C) and glomerular tuft (*P* < 0.0001) (Fig. 4D) when compared to their heterozygous littermates. Carnosine administration significantly reduced enlargement of Bowman’s capsule (*P* < 0.05) and space (*P* < 0.05). The surface area of the glomerular tuft was also lower in carnosine-administered mice; however, this did not reach statistical significance. In addition, enlargement of Bowman’s space positively correlated with glycemia (*P* < 0.0001), HbA1c (*P* = 0.001) and ACR (*P* < 0.001) (Fig. 4E).

### Carnosine restores glomerular ultrastructure without affecting podocyte number

**Figure 5:**
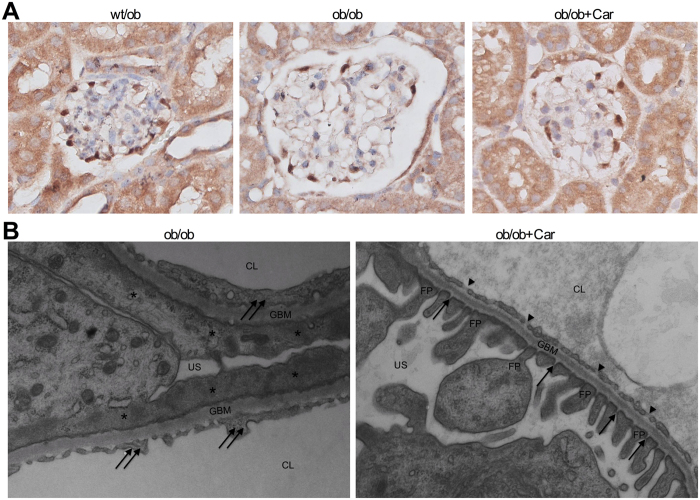
Effect of carnosine on glomerular podocytes, glomerular ultrastructure and mesangial matrix expansion. (**A**) Representative images of glomeruli immunostained for WT1 (and counterstained with hematoxylin) to visualize podocytes (brown) in *wt/ob, ob/ob* and *ob/ob* carnosine-supplemented mice. Podocyte loss observed in *ob/ob* mice was not prevented by carnosine treatment (quantification now shown). (**B**) Representative electron micrographs (30.000x) of glomeruli from *ob/ob* and *ob/ob* carnosine-supplemented mice. Untreated mice showed complete podocyte effacement (stars), GBM thickening and swelling of glomerular endothelial cells with loss of fenestrae (double arrows). Carnosine-treated mice displayed a normal capillary wall structure with intact podocyte foot processes, slit diaphragms (arrows) and endothelial fenestrae (arrowheads). FP: foot process; GBM: glomerular basement membrane; US: urinary space; CL: capillary lumen; Car: carnosine.

Since podocytes are critical cells for the maintenance of selectivity of the glomerular filtration barrier and become less in number with the onset of albuminuria in DN, we evaluated podocyte count by WT1 immunostaining (Fig. 5A). BTBR *ob/ob* mice showed a reduction in WT1-positive podocytes when compared to their heterozygous littermates (*P* < 0.001), which was not prevented by treatment with carnosine (data not shown). Immunostaining for cleaved caspase-3 showed a significant increase in apoptotic glomerular cells in both *ob/ob* groups as compared to *wt/ob* (data not shown).

At the ultrastructural level (Fig. 5B), all control *ob/ob* mice demonstrated global retraction and effacement of podocytes in their glomeruli. In addition, thickening of the GBM was observed. Glomerular endothelial cells from these mice had lost most of their fenestrae and had developed a swollen, vacuolized appearance. The ultrastructure of the glomerular walls from carnosine-treated mice showed none of these ultrastructural abnormalities. Their podocytes had normal slit pores, podocyte extensions and fenestrated endothelial cells.

Mesangial expansion is a key feature of human DN. We evaluated mesangial matrix expansion using PAS-stained sections. Kidney tissue was graded blindly on a scale of I to IV depending on the amount of mesangial matrix and glomerular sclerosis. BTBR *wt/ob* mice showed an average grade between I (0–10% mesangial matrix) and II (10–50%). Both BTBR *ob/ob* untreated and carnosine-treated mice showed an average grade between II (10–50%) and III (>50%). As expected, calculated average mesangial matrix expansion was higher in homozygous animals compared to their heterozygous littermates (*P* < 0.05). However, carnosine treatment did not prevent this increase in glomerular sclerosis (data not shown).

### Carnosine changes the molecular composition of mesangial sclerosis

**Figure 6:**
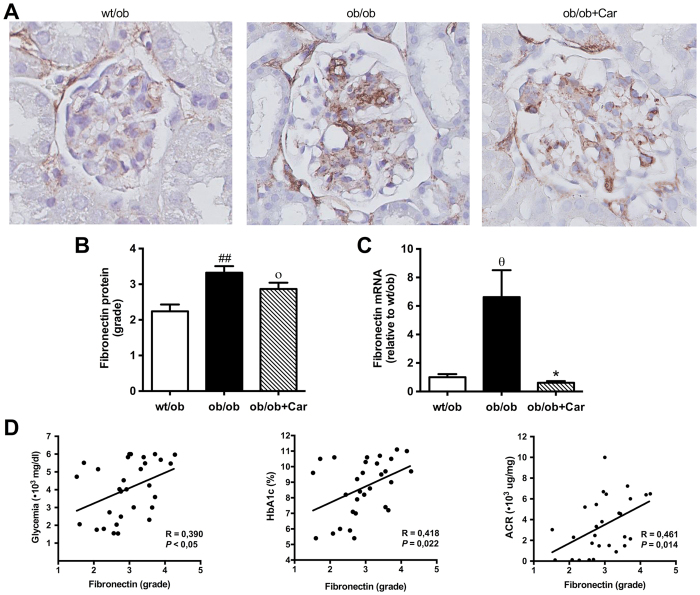
Effect of carnosine on fibronectin quantity in the mesangial matrix. (**A**) Representative images of glomeruli immunostained for fibronectin (brown) and counterstained with hematoxylin in *wt/ob, ob/ob* and *ob/ob* carnosine-supplemented mice. (**B**) The quantity of fibronectin protein was increased in *ob/ob* mice compared to *wt/ob* controls. A trend was observed towards lower fibronectin content in carnosine-treated animals. (**C**) Fibronectin mRNA expression (shown relative to the *wt/ob* group) was significantly decreased in carnosine-treated mice compared to *ob/ob* controls. (**D**) The quantity of fibronectin protein positively correlated with glycemia, HbA1c and albumin-creatinine ratio (ACR). Data represents means ± SEM. *P < 0.05, ^ο^*P* = 0.09, ^θ^*P* = 0.058 compared to *ob/ob* mice. ^##^*P* < 0.01 compared to *wt/ob* mice. Car: carnosine.

Since increased levels of several extracellular matrix (ECM) proteins are observed in early and late stages of DN, we investigated the molecular composition of the mesangial lesions by quantitative immunostaining. Indeed, immunostaining for fibronectin revealed that the glomerular lesions contained a substantial amount of fibronectin (Fig. 6A). Fibronectin content positively correlated with glycemia (*P* = 0.030), HbA1c (*P* = 0.022) and ACR (*P* = 0.014) (Fig. 6D). The quantity of fibronectin Protein was significantly higher in *ob/ob* mice when compared to their heterozygous littermates (*P* < 0.01) (Fig. 6B). We found a trend towards lower levels in the carnosine-administered mice, but this was not significant (*P* = 0.09) (Fig. 6B). mRNA expression analysis, however, revealed significantly decreased mRNA expression of fibronectin in the carnosine-supplemented mice relative to their untreated controls (*P* < 0.05) (Fig. 6C).

**Figure 7:**
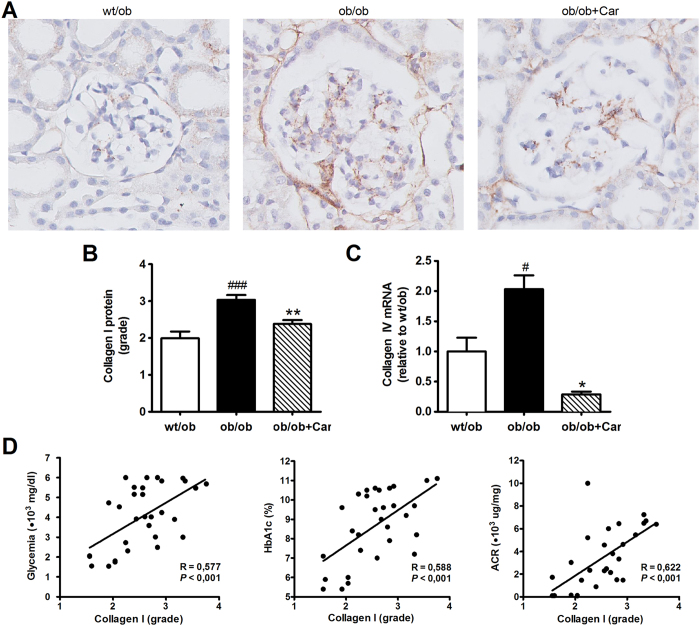
Effect of carnosine on the collagen quantity in the mesangial matrix. (**A**) Representative images of glomeruli immunostained for collagen I (brown) and counterstained with hematoxylin in *wt/ob, ob/ob* and *ob/ob* carnosine-supplemented mice. (**B**) The quantity of collagen I protein was significantly higher in *ob/ob* control animals compared to *wt/ob* mice, and attenuated in carnosine-treated animals. (**C**) Collagen IV mRNA expression (shown relative to the *wt/ob* group) was significantly decreased in carnosine-treated mice as compared to *ob/ob* controls. (**D**) The amount of collagen I positively correlated with glycemia, HbA1c and albumin-creatinine ratio (ACR). Data represents means ± SEM. *P < 0.05, **P < 0.01 compared to *ob/ob* mice. ^#^*P* < 0.05, ^###^*P* < 0.001 compared to *wt/ob* mice. Car: carnosine.

Immunostaining for collagen I showed that in addition to fibronectin, the glomerular lesions also contained collagen I (Fig. 7A). Collagen I content positively correlated with glycemia (*P* < 0.001), HbA1c (*P* < 0.001) and ACR (*P* < 0.001) (Fig. 7D). The quantity of collagen I was significantly increased in *ob/ob* mice when compared to their heterozygous littermates (*P* < 0.001) (Fig. 7B). Treatment with carnosine significantly diminished this increase in collagen I (*P* < 0.01) (Fig. 7B). Collagen I mRNA was expressed below the detection limit. Expression of collagen IV mRNA was over seven times lower in carnosine-supplemented mice as compared to untreated *ob/ob* mice (*P* = 0.01) (Fig. 7C).

### Carnosine and its acrolein adduct are increased in serum and urine of treated mice

Both carnosine and carnosine-propanal (=carnosine-acrolein adduct) were detected in the specimens of supplemented animals by means of nano flow liquid chromatography-Mass Spectometry. Analyte identity was confirmed by the accurate mass measurements and corresponding MSMS fragmentation patterns, which were largely superimposable with the spectra recently reported in a carnosine intervention study in humans^18^. All the predicted adducts deriving from the reaction of carnosine with HNE or glyoxal or methylglyoxal or MDA were undetectable.

**Figure 8:**
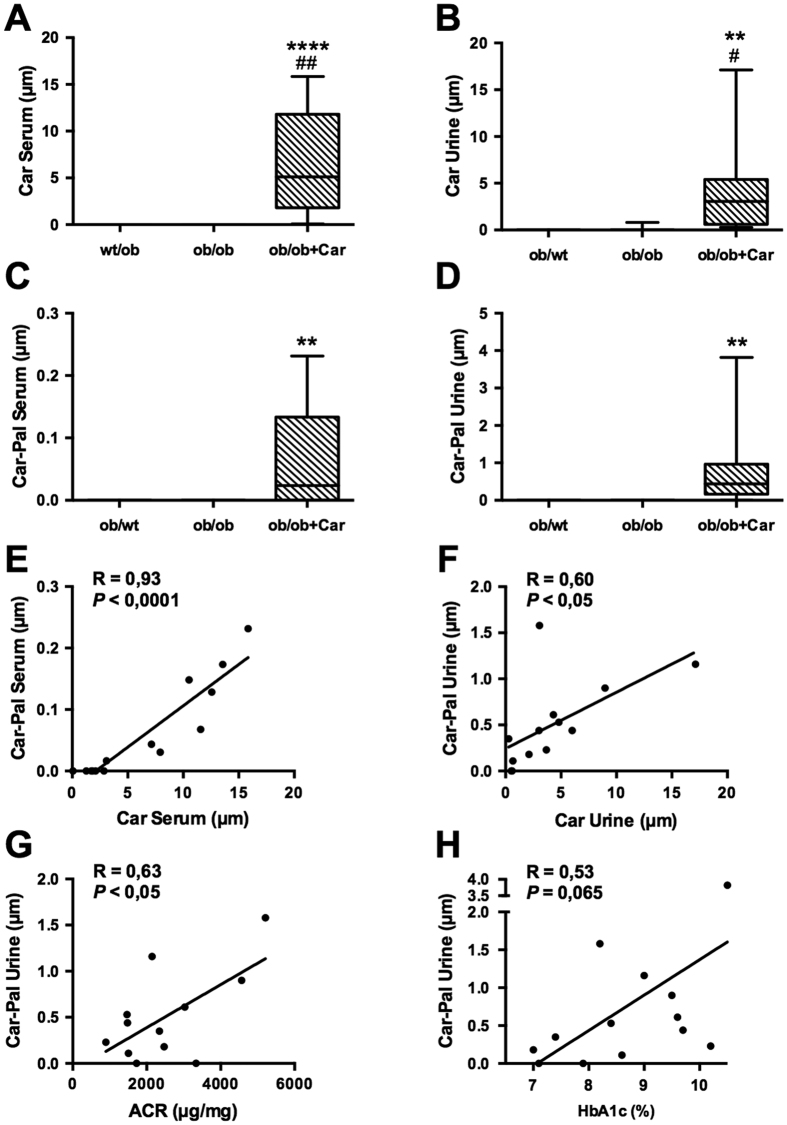
Carnosine and its acrolein adduct are increased in serum and urine of treated mice. (**A–D**) Carnosine and carnosine-propanal levels are significantly increased in the serum and urine of treated mice compared to *ob/ob* mice (data are depicted in a box plot with whiskers extended to minimum and maximum values). (**E,F**) Carnosine levels in serum and urine of treated mice significantly correlated with the respective adduct levels in the same specimen. (**G,H**) The ACR of treated animals significantly correlated with urinary carnosine-propanal. HbA1c levels showed a similar trend, but this correlation was not statistically significant (*P* = 0.065). ****P < 0.0001, **P < 0.01 compared to *ob/ob* mice. ^##^*P* < 0.01, ^#^*P* < 0.05 compared to *wt/ob* mice. Car: carnosine; Car-Pal: carnosine-propanal.

On the contrary of what has been observed in humans^18^, both carnosine and its adduct were not detectable in non-supplemented animals. Compared to *ob/ob* control animals, supplemented animals showed a significant increase of carnosine and carnosine-propanal adduct both in serum (P < 0.0001 and P < 0.01, respectively) and urine (*P* < 0.01 and *P* < 0.01, respectively) (Fig. 8A–D). The average carnosine concentration was higher in serum (6.57 μM and 4.24 μM, respectively), but the average carnosine-propanal concentration lower than in urine (0.06 μM and 0.74 μM, respectively). Both substances show a broader concentration range in urine (0.28–17.13 μM and 0.00–3,82 μM, respectively) than in serum (0.07–15.84 μM and 0.00–0.23 μM, respectively). Carnosine-propanal was below the serum LLOD for 6 supplemented animals out of 14, while in the urine only one supplemented animal had undetectable level of the adduct.

We found a significant correlation between carnosine and carnosine-propanal levels in both serum (*P* < 0.0001) and urine (*P* < 0.05) of treated animals (Fig. 8E,F). Interestingly, in treated animals the ACR also significantly correlated with carnosine-propanal levels (*P* < 0.05) (Fig. 8G). We found a similar trend for HbA1c levels, but this correlation did not reach statistical significance (*P* = 0.065) (Fig. 8H).

### Carnosine abrogates acrolein toxicity *in vitro* by inhibition of protein carbonylation

**Figure 9:**
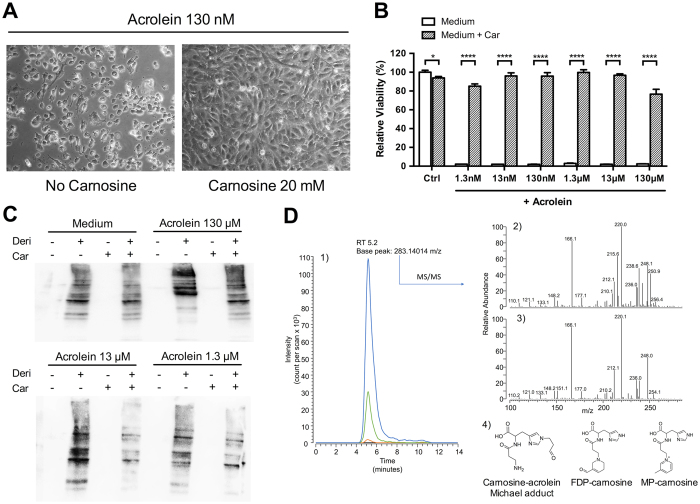
Carnosine abrogates acrolein toxicity *in vitro* by inhibition of protein carbonylation. (**A**) Incubation of HUVECs with 130 nM of acrolein for 24 hours led to cell death, which was completely reversed when co-incubated with carnosine. Analogous results were observed for acrolein concentrations as low as 1,3 nM. (**B**) Quantitative MTT analysis (n = 6 for each condition) revealed complete cytotoxicity for acrolein concentrations of 1.3 nM to 130 μM. Carnosine co-incubation restored cell viability. (**C**) Representative OxyBlot results of lysed cells after 24 hours incubation with acrolein in presence or absence of carnosine. Signal intensity of bands representing carbonylated proteins was increased in acrolein stimulated cells. This increase was diminished in presence of carnosine. Samples without addition of derivation solution served as negative controls. (**D**) Identification of carnosine-acrolein Michael adduct in cell supernatants. 1) Single ion chromatograms of cell supernatants containing a fixed amount of carnosine (20 mM) and no acrolein (black line), 0.13 mM acrolein (orange line), 1.3 mM acrolein (green line) or 13 mM acrolein (blue line) reconstituted by setting the filter ion at *m/z* 283.14014. 2) Tandem mass spectrum of the ion at *m/z* 283.14014 detected in cell supernatants. 3) Tandem mass spectrum of a standard carnosine-acrolein Michael adduct. 4) Carnosine-acrolein adducts detected in cell supernatants. *P < 0.01, ****P < 0.0001. Car: carnosine. Deri: derivation solution.

Based on the finding of significant carnosine-acrolein adduct generation in urine and sera of supplemented BTBR mice, we investigated if carnosine could affect viability of cells exposed to acrolein. To this end, HUVECs were subjected to various concentrations of acrolein for 24 hours in presence or absence of 20 mM of carnosine. At acrolein concentrations of 1.3 nM to 130 μM, light microscopy revealed significant toxicity as cells became round shaped and uniformly detached from the surface. Cells co-incubated with carnosine showed a completely normal morphology (Fig. 9A). At acrolein concentrations greater than 130 μM, toxicity was more and more masked due to an immediate fixation property of acrolein conserving cell morphology. To objectify the effect on cell viability, an MTT assay was carried out. It confirmed toxicity of acrolein over the whole concentration range used (1.3 nM – 130 μM), which was significantly abrogated by carnosine (*P* < 0.0001 for all concentrations) (Fig. 9B). To identify the mechanism behind this protection, we determined the amount of carbonylated proteins in cell lysates by using the OxyBlot technique. The resulting blots revealed enhanced signal intensity for carbonylated proteins in cells subjected to various concentrations of acrolein, which was clearly reduced in presence of carnosine (Fig. 9C). To prove that this inhibition of cellular protein carbonylation is due to a reaction of carnosine with acrolein, we assessed carnosine-acrolein adduct levels in cell supernatants by means of mass spectrometry (Fig. 9D). Carnosine-acrolein Michael adducts were identified only in the supernatants of cells incubated with both acrolein and carnosine. The presence of this adduct was confirmed by a chromatographic peak at 5.2 minutes for the single ion chromatogram extracted using the adduct theoretical *m/z* value as filter. Such a peak was detectable in samples incubated with an acrolein concentration above 0.13 mM, and showed a signal increase proportional to the amount of acrolein present in the cell media. Moreover, the spectrum base peak at 283.14008 had an *m/z* delta mass value of only +0.2 ppm from the expected adduct value. Structural confirmation was achieved by comparing the experimental fragmentation spectra of the product detected and a standard carnosine-acrolein Michael adduct. A similar response pattern was observed for adducts from multiple acrolein adduction, specifically for MP-carnosine which was detectable in dose-dependent-manner in the supernatants of cells incubated with acrolein at concentrations higher than 1.3 mM (data not shown).

## Discussion

In this study, we hypothesized that treatment with L-carnosine may ameliorate features of DN in the BTBR *ob/ob* model. Previous approaches have already indicated a beneficial effect of carnosine on several aspects of diabetic disease manifestation, however, they all failed to verify amelioration of DN at the morphological level. These limitations may be attributed to the mouse models used, as they all represent only early features of renal lesions. Moreover, STZ-mediated hyperglycemia did not allow an analysis of the effects of carnosine on β-cell function, since the insulin-producing cells were eliminated. We sought to overcome these obstacles by employing the BTBR *ob/ob* mouse model that results in the development of progressive renal pathology. The major outcomes of our investigation are as follows. Administration of carnosine led to elevated carnosine and carnosine-carbonyl adducts, improved both glucose metabolism and albuminuria, restored glomerular ultrastructure and hypertrophy and altered the molecular composition of the expanded mesangial ECM.

Carnosine-administered mice showed lower FPG throughout the whole observation period, which was confirmed by a lower HbA1c. Of note, in untreated *ob/ob* mice glycemia frequently exceeded the detection range (>600 mg/dl), so the differences in FPG may be even more pronounced in reality. Our results are consistent with other studies that also have reported glucose-lowering effects of carnosine^19^,^20^. In this study, improved glycemic control in treated mice was accompanied by twofold increased serum insulin levels when compared to non-treated *ob/ob* mice. The negative correlation of glycemia and serum insulin in treated mice suggests that elevation of insulin levels significantly contributes to the glucose-lowering effects of carnosine. In fact, insufficient islet compensation is a crucial factor for the predisposition of the BTBR strain to accelerated diabetes^13^. Moreover, it seems unlikely that hyperinsulinema in the carnosine-administered group is a consequence of reduced metabolic control, as a positive correlation would be expected in that case. Effects of carnosine on β-cell mass were described previously^21^, however, we could not confirm this as our histological analysis showed a focal expansion of β-cells with an extensive individual variation. As carnosine treated mice showed significantly higher C-peptide levels, it seems likely that carnosine had a stimulatory effect on insulin secretion, which can result from either direct internalization of carnosine in pancreatic β-cells or by actions of its two constituent amino acids β-alanine and histidine. Carnosine is a substrate of the proton-coupled peptide transporter 1 (PEPT1) found to be expressed in pancreatic tissue and may activate the insulin-signaling cascade^22^. In parallel, a considerable amount of carnosine is hydrolysed upon ingestion in enteral cells by carnosinase-2 (tissue carnosinase) and released into the circulation via amino acid transporters^23^. It is well known that amino acids are capable of stimulating glucose-dependent insulin response; in particular both histidine and alanine have been implicated to accelerate insulin secretion^24^,^25^,^26^. β-alanine could enter pancreatic cells together with depolarizing Na^+^-ions via the sodium-dependent TauT (taurine transporter) resulting in opening of voltage-activated L-type Ca^2+^ channels^27^,^28^. After internalization via LAT1 (L-type neutral amino acid transporter 1)^29^, histidine could be metabolised into α-ketoglutarate, which enters the Krebs cycle following complete oxidization to CO_2_ hereby raising the ATP/ADP ratio and subsequently insulin levels. Secondly, as a cationic amino acid, histidine can cause gating of voltage-sensitive Ca^2+^ channels as a result of primary depolarization of the plasma membrane. In hypothalamic neurons, carnosine can be metabolized into the neuronal transmitter histamine^30^ and bind to the presynaptic H_3_-receptor resulting in insulin secretion^31^,^32^.

Clinical diagnosis of DN is based on abnormal urinary albumin excretion. At week 24 of life, we measured a significant increase of the ACR in *ob/ob* animals, which was reduced more than twofold by carnosine treatment. In fact, after 10 weeks of life carnosine treatment stopped further progress of albuminuria. This was accompanied by a restored glomerular ultrastructure in carnosine-supplemented mice contrasting the advanced injury found in *ob/ob* control mice, including podocyte effacement, GBM thickening and endothelial dysfunction. On the contrary, the WT1-staining did not show any differences between control and carnosine treated animals. Importantly, recent studies identified podocyte injury rather than podocyte loss as the primary structural factor responsible for loss of glomerular selectivity^33^. Likewise, the decrease in WT-1 positive cells found at week 24 (approximately two/glomerulus) is unlikely to explain the 50x difference in proteinuria between *ob/ob* and *wt/ob* mice, whereas this difference is very consistent with the pronounced podocyte injury found ultrastructurally.

To our knowledge, this is the first study to reveal an impact of carnosine on glomerular hypertrophy. BTBR *ob/ob* mice showed an increased glomerular hypertrophy as characterized by a 2.2 times increased Bowman’s capsule surface area. A recent patient study illustrated that obese subjects exhibiting proteinuria displayed a twofold increase in Bowman’s space volume when compared to lean control subjects^34^. In our mouse model, carnosine was able to significantly reduce the size of the renal capsule mainly via a decrease in the surface area of Bowman’s space. Since we ruled out shrinkage of glomerular capillaries, this reduction in size is most likely a consequence of diminished glomerular hyperfiltration. Indeed, the presence of hyperfiltration in the BTBR *ob/ob* mouse has already been described recently^16^. Glomerular hyperfiltration is considered one of the key events in the development of DN^35^. The clinical importance becomes apparent when realizing that ACE-inhibitors still are the only medication for diabetic patients with nephropathy in order to delay progression. A very recent study on D-carnosine-octylester (a bioavailable carnosine-derivate) in STZ treated diabetic ApoE-null mice displayed similar findings with reduced renal hypertrophy and albuminuria, but no effect on cholesterol^36^. As glycemic control remained unaffected in this model, we speculate that at least part of the renoprotection is mediated by carnosine, whereas the insulinotropic effect may be mediated by histamine and β-alanine.

Carnosine treatment reduced the deposition of fibronectin and collagen I by mesangial cells and mRNA expression of fibronectin and collagen IV. An *in vivo* effect of carnosine on the composition of the ECM has never been described before. This may be due to the fact, that BTBR *ob/ob* mice exert strongly increased accumulations of mesangial matrix even when compared to the C57BLKS/J *db/db* mouse model^11^. The expression of collagen I mRNA was below the detection limit, since it is only expressed in advanced glomerulosclerosis^37^.

Recently, a growing interest emerged in the ability of carnosine to quench reactive carbonyl species through Schiff base formation^38^,^39^,^40^. Acrolein is the most reactive of the α,β-unsaturated aldehydes and has been associated with diabetic nephropathy^41^,^42^. In this study, oral carnosine supplementation not only led to increased carnosine, but also carnosine-carbonyl levels. The good correlation between the concentration of carnosine and carnosine adduct in serum and urine suggests that supplemented carnosine reacted with endogenous acrolein to generate stable adducts which were excreted in urine. Subsequent *in vitro* experiments with HUVECs evinced a complete reversal of acrolein toxicity in presence of carnosine, which was paralleled by a significant reduction of cellular protein carbonylation as assessed by the OxyBlot technique. Mass spectrometry confirmed neutralization of the reactive aldehyde in cell supernatants. The fact, that no adducts were found in supernatants from cells exposed to acrolein concentrations lower than 130 μM is due to sensitivity limitations of the assay. As carbonylation results in protein dysfunction and the formation of AGEs implicated in diabetes, detoxification of the reactive precursors by carnosine may be a central mechanism to the protective effects observed in BTBR mice^43^. Evidence for this hypothesis comes from the recent finding, that urinary acrolein metabolites are associated with diabetes and insulin resistance^44^.

Though it is difficult to differentiate direct renoprotective effects from those secondary to improved glycemia as a consequence of elevated insulin levels, we assume that direct actions play an essential role in the findings of this study and need to be considered separately. In particular, the profound differences in albuminuria and at the ultrastructural level indicate mechanisms beyond attenuation of glycemic control, one of which may be the neutralization of reactive carbonyl species as demonstrated *in vivo* and *in vitro*. In fact, there is a considerable amount of evidence on renoprotective effects of carnosine. Studies report carnosine-induced diminishment of fibronectin and collagen type VI production in podocytes and normalization of TGF-beta production in mesangial cells incubated with high glucose medium^6^,^45^. Other mechanisms include the inhibition of lipid peroxidation, glycation and oxidative modifications of proteins by carnosine^46^,^47^. Of note, methylglyoxal, an endogenous metabolite accumulating in diabetes, can be neutralized by carnosine^48^. Even anti-ageing effects in T cells extending the lifespan under physiological oxygen tension have been described^49^. Additional supportive *in vivo* data comes e.g. from ischemia/reperfusion models^50^,^51^ and STZ-rendered diabetic mice with renal improvement despite unaffected glycemic control^10^,^36^,^52^.

A major limitation of this study results from differences between murine and human carnosine metabolism. In contrast to humans, rodents do not secrete carnosinase-1 in the circulation. However, this enzyme very efficiently breaks down carnosine into its two components β-alanine and L-histidine. Therefore, in humans oral supplementation only leads to minimal increases^53^. However, we have recently shown that the human kidney is able to synthesize its own carnosine after ingestion of the individual amino acids^54^. Since in mice higher systemic concentrations can be achieved, the translational significance using a rodent model may be limited. To address this obstacle, we currently establish a BTBR *ob/ob* mouse model transgenic for human CN1, which will be subject of a separate report. Further studies on carnosine in diabetes are warranted to investigate the mechanisms relevant for insulin sensitivity, glucose tolerance and possible implications on the inflammatory phenotype.

In conclusion, we have demonstrated that treatment with carnosine is able to attenuate both glucose metabolism and albuminuria in more advanced stages of DN. This was mirrored morphologically in restored glomerular ultrastructure, reduced glomerular hypertrophy and modifications of the ECM composition. Therefore, carnosine could be a promising therapeutic treatment modality to ameliorate both diabetes and the development of DN.

## Methods

### Experimental design

All experiments and methods were performed in accordance with relevant guidelines and regulations. All animal procedures were approved by the Regierungspräsidium Karlsruhe (AZ 35-9185.81/G-119/11). Six-week old male BTBR mice were purchased from Jackson Laboratories (Bar Harbor, ME, USA) and divided into three groups: BTBR *wt/ob* (n = 5), BTBR *ob/ob* (n = 15) and BTBR *ob/ob* supplemented with 45 mg/kg body weight L-carnosine (n = 15) dissolved in drinking water (4 mM; Flamma, Italy) as previously described^21^. BTBR *wt/ob* mice served as a negative control of the BTBR *ob/ob* mouse model for DN, since these mice are not leptin deficient. Mice were housed at 22 °C in a 12 h light/dark cycle and fed regular chow ad libitum. Drinking bottles containing carnosine were replaced at least every third day to guarantee stability. Body weight, and fasting plasma glucose (FPG) were assessed weekly at the beginning of the light cycle after a 5-hours fasting period using an OneTouch Verio IQ blood glucose meter (LifeScan, Milpitas, CA). Glycated hemoglobin (HbA1c) was measured using the in2it A1C system (Bio-Rad, Hercules, CA). At week 24 of age (after 18 weeks of treatment), blood samples were collected from the orbital plexus under anesthesia. Proteinuria was determined using the ACR instead of a 24-hour total urinary albumin excretion since the BTBR strain is very susceptible to stress and due to recent studies showing equivalence of both measures^55^. To obtain morning spot urine samples, animals were placed in metabolic cages for two hours at the beginning of the light cycle and received only water but no food. Creatinine, blood urea nitrogen and cholesterol concentrations were measured using a Cobas^®^ C311 Autoanalyzer (Roche Diagnostics, Indianapolis, USA) according to the manufacturer’s procedures. Urinary albumin, insulin, glucagon, C-reactive protein and C-Peptide were determined by ELISA.

### Histological analysis

After 18 weeks of treatment, mice were sacrificed by vascular perfusion fixation through the aorta with 4% paraformaldehyde under ketamine/xylazine anesthesia. Kidneys were isolated and weighed. A subset of kidneys was also preserved cryogenically. Tissues fixed with paraformaldehyde were embedded in paraffin, cut in 4 μm sections, deparaffinized with xylol and dehydrated using an ethanol gradient. Sections were stained with periodic acid-Schiff (PAS) and hematoxylin and eosin (H&E). Stained slides were digitalized with Philips Digital Pathology Solutions (Philips Electronics) for morphological measurement. PAS-stained kidney tissue was graded blindly on a scale of I to IV depending on the amount of mesangial matrix: I, 0–10%; II, 10–50%; III, >50%; IV: global glomerular sclerosis. For electron microscopy, 1-mm-thick slices were fixed with 3% glutaraldehyde in 0,1 M CaCo buffer. After processing and sectioning according to standard protocols, two EM-samples of each diabetic group were examined blindly.

### Immunohistochemistry

Sections were deparaffinized and rehydrated as described above. For immunostainings of Wilms tumor protein (WT1), cleaved caspase-3 and collagen I, antigen retrieval was performed using Tris/EDTA buffer (pH 9.0, 10 mM; 20 min, 100 °C), citrate buffer (pH 6.0, 10 mM; 20 min, 100 °C) or pepsin (0.4% in 0.1 HCL; 15 min, 37 °C), respectively. Endogenous peroxidase was blocked with 0.12% H_2_O_2_ in Milli-Q (20 min, RT), followed by incubation with primary antibody diluted in 1% BSA in PBS. Antibodies used were (1) rabbit anti-fibronectin (Sigma-Aldrich, St.Louis, MO); (2) rabbit anti-collagen I (AbD Serotec, Puchheim, Germany); (3) rabbit anti-WT1 (Santa Cruz Biotechnology, Santa Cruz, CA); and (4) rabbit anti-cleaved caspase-3 (Cell Signaling Technology, Danvers, MA). Negative controls for immunohistochemistry included normal sera of the same species as the primary antibody. The immunoreactions were visualized with 3,3-diaminobenzidine (Dako), counterstained with hematoxylin, dehydrated, and mounted. Cell numbers were determined by randomly analyzing 25 glomeruli of each experimental animal. Sections stained for fibronectin and collagen I were digitalized and graded blindly on a scale of I to V depending on the staining intensity: I, none; II, minute; III, moderate; IV, high; V, very high.

### RNA isolation, cDNA synthesis and quantitative RT-PCR

TRIzol RNA isolation reagent (Thermo Fisher Scientific) was added to the subset of cryogenically preserved kidney tissue. The samples were incubated (3 min) with chloroform and centrifuged (13.000 rpm; 10 min, 4 °C). The supernatant was isolated and RNA was precipitated using isopropanol (20 min, RT) and centrifuged (13.000 rpm; 10 min, 4 °C). The obtained pellet was washed with 75% ethanol and centrifuged (13.000 rpm; 10 min, 4 °C). Thereafter, the pellet was dried, dissolved in nuclease-free water and incubated for 10 min at 60 °C. The RNA concentration and quality was determined with a Nanodrop spectrophotometer (Thermo Scientific). Total RNA was reverse-transcribed with AMV-RT (Roche). The RT-PCR was performed with a SYBR Green kit (Bio-Rad) on a CFX96 RT-PCR Detection System (Bio-Rad). Primers used for detection of mouse fibronectin were GGCAGGCTCAGCAAATCG (forward) and CATAGCAGGTACAAACCAGGG (reverse), and for detection of mouse collagen IV CGTCTCTGCTGGTCCCCT (forward) and GGCAAGCCTCTTTCTCCCTT (reverse). mRNA expression of genes of interest was compared to mRNA expression of housekeeping genes (*HPRT* and *GAPDH*).

### Nano flow Liquid Chromatography-Mass Spectometry

Before analysis, urine samples were diluted ten fold with 1% (v/v) aqueous perfluoropentanoic acid (mass spectrometry grade, Sigma-Aldrich, Milan, Italy) containing 1 μm of the internal standard tyrosyl-histidine (Flamma S.p.A., Chignolo d’Isola, Bergamo, Italy). Samples were centrifuged at 14.000 g for 10 minutes to precipitate insoluble particles. Serum samples were deproteinized by adding acetonitrile (LCMS grade, Sigma-Aldrich, Milan, Italy) up to 80% (v/v) and kept at 5 °C for 10 minutes. The samples were then spun as described above and the supernatants collected and evaporated at room temperature by a RVC 2–18 rotational vacuum concentrator. Dried residues were re-dissolved in 1% (v/v) aqueous PFPA containing the internal standard to have a ten-fold final dilution of serum.

Calibration samples were prepared in triplicates by spiking carnosine in blank matrices at the following final concentrations: 0.5, 1, 10, 50, 100, 500 μM for urine and 0.5, 1, 10, 50, 100 μM for serum. Calibration curves were built by least square linear regression plotting the nominal concentration of the analytes (x axis) versus the analytes/internal standard peak area ratio (y axis). One calibration curve was prepared using commercially available rodent serum to calculate carnosine concentrations in serum samples. Since rodent urine was not available commercially, a second calibration curve was prepared using human urine from a vegan volunteer to calculate urinary carnosine concentrations. A vegan subject was recruited since background carnosine is detectable in human urine, but it was reported that a meat-free diet significantly reduces carnosine urinary concentration^18^.

Before preparing calibration curves, blank matrices were checked. Carnosine adducts were not detected in any matrix, while carnosine was detectable only in blank urine at a concentration below the LLOQ of the analytical method.

The analytic platform was composed of a Dionex UltiMate3000 nano-flow LC system connected to an LTQ-Orbitrap XL mass spectrometer through a nanoelectrospray ionization source (NSI), assembled by a Finnigan NSI-1 dynamic probe equipped with a stainless steel LC/MS emitter (5 cm length, O.D. 150 μm, I.D. 30 μm, Thermo Fisher, Rodano, MI, Italy).

The chromatographic separation was performed at 30 °C at a flow rate of 200 nL/min by using a binary gradient of mobile phase A (water containing 0.1% formic acid) and mobile phase B (acetonitrile containing 0.1% formic acid). Sample loading was performed at 30 °C at a flow rate of 5 μL/min by using water containing 0.1% PFPA as mobile phase. Sample loading and cleanup/desalting was performed into an Acclaim^®^ PepMap 100 trap column (100 μm × 2 cm, C18, 5 μm, 100 Å, Thermo Fisher Scientific, Rodano, MI, Italy), while an Acclaim^®^ PepMap 100 (100 μm × 15 cm, C18, 5 μm, 100 Å, Thermo Fisher Scientific, Rodano, MI, Italy) was used as analytical column.

The flow was kept diverted to the waste for 4 minutes to allow sample loading/cleanup, afterwards the trap column was switched to be in line with the analytical column. The elution of the analytes was performed by a multi-step gradient starting with a 5-minute isocratic flow at 1% mobile phase B (99% mobile phase A), followed by a 12-minute linear gradient (1–99% mobile phase B, 99–1% mobile phase A) and eventually a 10-minute program for washing the column with 99% B and re-equilibrating the system to the initial conditions.

The flow coming from the column was sprayed directly into the mass spectrometer by the NSI source without using auxiliary gas. The source was operating in positive ion mode as follows: spray voltage 1.7 kV, capillary temperature 200 °C and capillary voltage 35 V.

During the chromatographic separation the MS spectra were acquired in profile mode by the Orbitrap using the following settings: scan range 200–600 m/z, 5 × 10^5^ ions per scan, maximum inject time was set to 500 ms, resolution was set to 30000 (FWHM at m/z 400).

Data dependent feature was enabled to collect MS/MS spectra for the six most intense ions of each scan exceeding a count of 5 × 10^4^, using the linear ion trap and the following settings: centroid mode, precursor ions isolation width of 3 m/z, 1 × 10^4^ ions per scan, maximum inject time was set to 1000 milliseconds. Collision energy of 35% and an activation time of 30 milliseconds were used for CID fragmentation with wideband option enabled.

Dynamic exclusion was enabled to reduce redundant MS/MS spectra acquisition using default settings and an exclusion window of 20 ppm. Charge state screening and monoisotopic precursor selection were enabled to allow fragmentation of singly charged ions only. Lock mass option was enabled to provide a real time internal mass calibration during the analysis using as reference a list of 20 abundant and known background signals already reported by Keller *et al*. as common air contaminants in mass spectrometry^56^. Instrument control was provided by the software Xcalibur and Chromeleon Xpress.

Carnosine and carnosine adducts were identified based on accurate mass measurements and corresponding CID fragmentation patterns. Peak areas were calculated from extracted single ion chromatograms (SICs), centered within a tolerance window of 10 ppm from the theoretical mass values of internal standard, carnosine and carnosine adducts.

Carnosine concentration was calculated using the corresponding calibration curves for serum and urine. Adduct concentration was determined by the following equation:


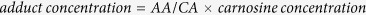


(AA = aduct peak area, CA = carnosine peak area).

### Cell culture and isolation

Human umbilical vein endothelial cells (HUVEC) were isolated from fresh umbilical cords. The cells were cultured in essential growth medium for endothelial cells in gelatine-coated culture flasks at 37 °C, 95% relative humidity and 5% CO_2_. Confluent monolayers were passaged by trypsin 0.025%/EDTA 0.01%. For MTT assays, HUVEC were seeded in 96-well plates at a density of 3 × 10^4^ cells/well and grown until confluence. HUVECs were used in passages 3–5 and all results confirmed in at least three independent experiments.

### MTT (3-(4,5-dimethylthiazol-2-yl)-2,5-diphenyltetrazolium bromide) tetrazolium reduction assay

HUVEC were stimulated for 24 hours with different concentrations of acrolein (1.3 nM–130 μM) in the absence or presence of 20 mM of carnosine. Cells maintained in normal culture medium or in culture medium to which 20 mM of carnosine was added served as control. Hereafter, all supernatants were aspirated and replaced by 200 μL of MTT reagent (0.5 mg/ml) and the plates were incubated for 6 hours at 37 °C, 95% relative humidity and 5% CO_2_. After conversion of MTT into a purple coloured formazan product in viable cells, the plates were aspirated and wells were filled with a solubilization solution containing 4 parts DMSO, 4 parts 10% w/v SDS and 2 parts of PBS/acetic acid in a final concentration of 1.2% v/v. The plates were incubated overnight and recorded at 560 nm with reference wavelength at 670 nm. Relative cell viability was assessed as ratio of OD sample/OD control, both background-corrected.

### Protein extraction and OxyBlot

HUVEC were resuspended in lysis buffer (10 mM Tris–HCl, 150 mM NaCl, 5 mM EDTA, 1% Triton X-100, 0.5% sodium deoxycholate, 1 μM dithiothreitol (DTT), proteinase inhibitor cocktail and phosphatase inhibitor). Protein concentrations were measured using Coomassie-Reagent (Pierce, Rockford, USA). Samples (5 μg protein extract) were subjected to the OxyBlot protocol (Merck, Darmstadt, Germany) according to the manufacturer’s instructions, loaded and separated on 10% SDS-polyacrylamide gels followed by semi-dry blotting onto PVDF membranes (Roche, Mannheim, Germany). The membranes were incubated with 5% w/v non-fat dry milk or bovine serum albumin in TBS/Tween 0.1% to block unspecific background staining and hereafter incubated for one hour at 4 °C with primary antibody, specific to the carbonyl group derivatization product (2,4-dinitrophenylhydrazone). Subsequently, the membranes were thoroughly washed with TBS-Tween 0.1% and incubated with the appropriate horseradish peroxidase conjugated secondary antibody, followed by five times wash in TBS/Tween 0.1%. Proteins were visualized using enhanced chemoluminescence technology, according to the manufacturer’s instructions (Pierce, Rockford, IL, USA).

### Carnosine-acrolein adduct identification in cell supernatants

Before analysis, supernatants from HUVECs exposed to various acrolein concentrations in absence or presence of carnosine were centrifuged for 10 minutes at 14000 g, filtered through 0.45 μm membrane and diluted 200 fold with 0.1% aqueous formic acid.

Chromatographic separations were performed by a Hypersil GOLD HILIC column (150 × 2.1 mm, 3 μm) using an UltiMate3000 chromatograph (Thermo Scientific, Rodano, MI, Italy). The separation program started with a 2 μL partial loop injection of diluted supernatants. The analytes of interest were then eluted by a binary gradient using pure acetonitrile as mobile phase A and 0.1% aqueous formic acid as mobile phase B. The elution program started with a one-minute isocratic flow at 10% mobile phase B, followed by a linear ramp up to 40% mobile phase B in 4 minutes which was then kept constant for an additional 4 minutes before a five-minute program to re-equilibrate the system to the initial condition.

The flow coming from the column was sprayed directly into an LTQ Orbitrap XL mass spectrometer by a Finnigan Ion Max electrospray interface (Thermo Scientific, Rodano, MI, Italy). Nebulization was achieved using a spray voltage of 5 kV, a capillary temperature of 275 °C and 35 units of sheath gas. During the elution, the detector scanned positive ion mass spectra in a 200–600 m/z range at a resolution of 100000 (FWHM at 400 m/z). Tandem mass spectra were acquired in ion trap for any ion within 5 ppm of the predicted *m/z* values for the main carnosine-acrolein adducts, namely carnosine-acrolein Michael adduct (283.140082 m/z), carnosine-acrolein Schiff base (*m/z* 265.129517), N-terminus-(3-formyl-3,4-dehydropiperidino)carnosine (FDP-carnosine, *m/z* 321.155732) and N-terminus-(3methylpyridinium)carnosine (MP-carnosine, *m/z* 303.145167).

Full instrument control and extraction of peak areas used for quantitation were provided by Xcalibur and Chromeleon Xpress software (version 2.0.7, Thermo Fisher Scientific, Rodano, MI, Italy).

### Statistical analysis

All data are expressed as mean ± SEM. Statistical analysis was performed using one-way ANOVA. Significant omnibus results were followed by Fisher’s LSD test, which is more powerful than alternatives when testing differences among three groups^57^. To compare the means of two groups, quantitative analysis was carried out using unpaired, two-tailed t-test. Two-tailed, Pearson’s correlation was performed to identify correlations using SPSS 20 software package for Windows (SPSS, Chicago, IL). Differences were considered statistically significant at *P* < 0.05.

## Additional Information

**How to cite this article**: Albrecht, T. *et al*. Carnosine Attenuates the Development of both Type 2 Diabetes and Diabetic Nephropathy in BTBR *ob/ob* Mice. *Sci. Rep.* **7**, 44492; doi: 10.1038/srep44492 (2017).

**Publisher's note:** Springer Nature remains neutral with regard to jurisdictional claims in published maps and institutional affiliations.
